# Enhancing Molecular Aggregations by Intermolecular Hydrogen Bonds to Develop Phosphorescent Emitters for High‐Performance Near‐Infrared OLEDs

**DOI:** 10.1002/advs.201801930

**Published:** 2019-02-07

**Authors:** Xiaolong Yang, Haoran Guo, Xianbin Xu, Yuanhui Sun, Guijiang Zhou, Wei Ma, Zhaoxin Wu

**Affiliations:** ^1^ MOE Key Laboratory for Nonequilibrium Synthesis and Modulation of Condensed Matter Department of Chemistry School of Science Xi'an Jiaotong University Xi'an 710049 P. R. China; ^2^ State Key Laboratory for Mechanical Behavior of Materials Xi'an Jiaotong University Xi'an 710049 China; ^3^ Key Laboratory for Physical Electronics and Devices of the Ministry of Education Faculty of Electronic and Information Engineering Xi'an Jiaotong University Xi'an 710049 P. R. China

**Keywords:** hydrogen bonds, molecular aggregation, near‐infrared emission, organic light‐emitting devices, Pt(II) complexes

## Abstract

Phosphorescent near‐infrared (NIR) organic light‐emitting devices (OLEDs) have drawn increasing attention for their promising applications in the fields such as photodynamic therapy and night‐vision readable displays. Here, three simple phosphorescent Pt(II) complexes are synthesized, and their intermolecular interactions are investigated in crystals and neat films by X‐ray single crystal diffraction and grazing‐incidence wide‐angle X‐ray scattering, respectively. The photophysical properties, molecular aggregation (including Pt–Pt interaction), molecular packing orientation, and electron transport ability are all influenced by the strong intermolecular hydrogen bonds. Consequently, the nondoped OLEDs based on **tBu‐Pt** and **F‐Pt** show electroluminescent emissions in NIR region with the highest external quantum efficiencies of 13.9% and 16.7%, respectively.

Since the seminal work of Tang and VanSlyke, organic light‐emitting devices (OLEDs) have attracted significant interest from both academia and commercial communities for their application in flexible displays and solid lightings.^1^, ^2^, ^3^ To date, the most efficient blue‐, green‐, orange‐, and red‐emitting OLEDs have all achieved external quantum efficiencies (EQEs) above 30%.^4^, ^5^, ^6^, ^7^, ^8^, ^9^, ^10^, ^11^, ^12^, ^13^ Inspired by the successful development of visible light‐emitting OLEDs, the research on near‐infrared (NIR) OLEDs with an emission peak wavelength beyond 700 nm has drawn increasing attention because of their special applications in photodynamic therapy, night‐vision readable displays, and bio‐imaging.^14^, ^15^, ^16^, ^17^ However, for a long time, the efficiencies of NIR OLEDs are much lower than those of visible light‐emitting OLEDs because of the rarity of highly efficient NIR emitters. Although pure organic molecules,^18^, ^19^, ^20^, ^21^, ^22^ conjugated polymers^23^, ^24^, ^25^ or organometallic complexes based on osmium(II),^26^, ^27^ iridium(III),^28^, ^29^, ^30^ lanthanide,^31^, ^32^ and platinum(II)^33^, ^34^ have been used as NIR emitters. Most of NIR emitters are designed by extending the conjugated system and/or adopting donor‐acceptor structure, which increases the complexity of synthetic process, and thereby leads to the rarity of NIR emitters. Even more unfortunately, these NIR emitters show low photoluminescence quantum yields (PLQYs) because of the energy gap law which indicates that the PLQY reduces with decreasing energy gap.^35^ Therefore, to the best of our knowledge, the highest EQEs of NIR OLEDs with emission peak over 700 nm based on the pure organic molecules, conjugated polymers, Os(II) complexes, and Ir(III) complexes are 9.74%, 1.15%, 2.7%, and 4.5%, respectively.^27^, ^30^, ^36^, ^37^ However, the NIR OLEDs based on Pt(II) complexes can display higher EQEs. The device using a tetrabenzoporphyrin Pt(II) complex with extended conjugation as the emitter displayed NIR emission with an EQE of 9.2%.^38^ Two more efficient NIR OLEDs were fabricated based on terdentate cyclometallated Pt(II) complexes with the EQEs of 10.5% and 14.5%, respectively.^39^, ^40^ Very recently, based on 2‐pyrazinyl pyrazolate Pt(II) complexes, Chi and co‐workers reported extremely efficient NIR OLEDs achieving the EQEs up to 24 ± 1% without any light out‐coupling, which represents a milestone in the field of NIR OLEDs.^41^

The success of Pt(II) complexes in the development of high‐performance NIR OLEDs can be attributed to several aspects. Generally, Pt(II) complexes with a square‐planar structure usually tend to show strong intermolecular interactions in high concentrations or neat films, which will cause the formation of excimers or the metal–metal‐to‐ligand charge transfer (MMLCT) transition, and thereby lead to low energy emissions in the NIR region without extending the conjugated system and/or adopting donor‐acceptor structure.^39^, ^40^, ^41^ Besides, as phosphorescent emitters, Pt(II) complexes can harvest both singlet and triplet excitons in OLEDs to reach 100% internal quantum efficiency theoretically.^42^ Most importantly, unlike common phosphorescent emitters which suffer decreased emission efficiencies due to triplet–triplet annihilation (TTA) in high concentrations,^43^ Pt(II) complexes with suitable ligands can remain or even show increased PLQYs up to nearly unity in neat films.^9^, ^41^ However, suitable Pt(II) complexes with simple chemical structures and synthetic processes for high‐performance NIR OLEDs are still very rare. In this study, we report three very simple pyrimidine‐based Pt(II) complexes prepared from only two synthetic steps for highly efficient NIR OLEDs. The key point is to enhance molecular aggregations and Pt–Pt interactions by hydrogen bonds, thus the emissions can be easily shifted to low energy region. Consequently, the neat films of these Pt(II) complexes could show bright emissions in deep red/NIR region with high PLQYs up to 0.74. With a conventional device structure, the nondoped OLEDs can show NIR emissions peaking at 708 and 724 nm with the maximum EQEs of 13.9% and 16.7%, respectively, which are among the highest efficiencies ever reported for NIR OLEDs.^39^, ^40^, ^41^

**Scheme** **1** illustrates the synthetic routes for Pt(II) complexes **H‐Pt**, **tBu‐Pt**, and **F‐Pt**. The ligands were obtained by treating the 2‐chloropyrimidine with respective boronic acid compounds through Suzuki–Miyaura coupling reactions. Then, the complexes were prepared in high yields by the conventional method reported previously.^44^ The single crystals of these Pt(II) complexes were cultivated by slowly evaporating the solvents from CHCl_3_/hexane solutions. X‐ray diffraction results revealed that the complex molecules packed as head‐to‐tail dimers in crystals (**Figure** **1**). Some related key data is listed in Table S1 in the Supporting Information. As shown in Figure 1, the closest Pt–Pt distances (*d*_Pt–Pt_) in the crystals of **H‐Pt** and **tBu‐Pt** are 4.663(4) and 4.8920(8) Å, respectively, indicating the absence of metal–metal interactions. However, the dimers have a vertical plane‐to‐plane separation (*d*_π–π_) of ≈3.31 Å for **H‐Pt** and ≈3.22 Å for **tBu‐Pt**. Besides, the slippage distance between the π system of each molecule within the dimer of **H‐Pt** and **tBu‐Pt** is comparable, since the angles between the centroid distance and the vertical distance in **H‐Pt** and **tBu‐Pt** dimers are calculated to be 49.8° and 49.3°, respectively. These results indicated the existence of strong molecular aggregation or π–π interactions in **H‐Pt** and **tBu‐Pt** dimers. The *d*_Pt–Pt_ in the crystal of **F‐Pt** is only 3.3752(7) Å, which is indicative of strong metal‐metal interactions. In addition, the vertical plane‐to‐plane separation in **F‐Pt** is shorten to ≈3.17 Å, and the angle between the centroid distance and the vertical distance in the **F‐Pt** dimer is notably reduced to 28.5°, suggesting the significantly enhanced π–π interactions. Considering the similar molecular structure and size of these Pt(II) complexes, the strength of π–π interactions in the order of **H‐Pt** < **tBu‐Pt** < **F‐Pt** can be explained by hydrogen bonds. As depicted in **Figure** **2**, due to the unchelated N atom in the pyrimidine ring, both intramolecular hydrogen bonds (indicated by violet dashed lines) and intermolecular hydrogen bonds can be found in the crystals of **H‐Pt** and **tBu‐Pt**. In the crystal of **H‐Pt**, the intermolecular hydrogen bonds with a bond length of ≈2.9825 Å only exist between the molecules within dimers (indicated by blue dashed lines). However, besides the intermolecular hydrogen bonds within dimers (≈2.9765 Å), intermolecular hydrogen bonds with much shorter bond length of ≈2.6502 Å can be found between the neighboring dimers (indicated by red dashed lines) in the crystal of **tBu‐Pt**. In the crystal of **F‐Pt**, besides the strong intermolecular hydrogen bonds (≈2.6286 Å) induced by unchelated N atoms, much stronger intermolecular hydrogen bonds with a shorter bond length of ≈2.5782 Å are induced by F atoms, which will significantly enhance the molecular aggregations to influence their properties.

**Scheme 1:**
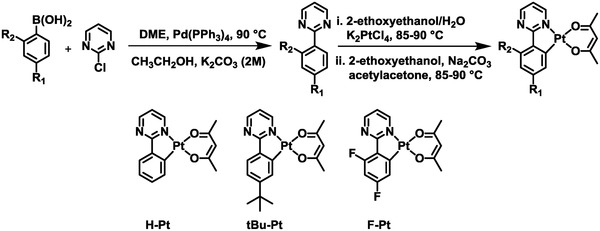
The synthetic routes and chemical structures of **H‐Pt**, **tBu‐Pt**, and **F‐Pt**.

**Figure 1:**
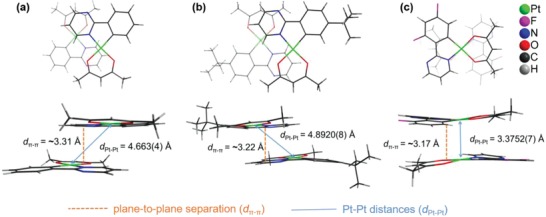
Top and side views of the Pt(II) complex dimer in crystals: a) **H‐Pt**, b) **tBu‐Pt**, and c) **F‐Pt**.

**Figure 2:**
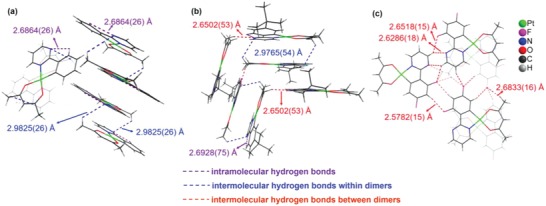
Hydrogen bonds in the crystals: a) **H‐Pt**, b) **tBu‐Pt**, and c) **F‐Pt**.

In essence, hydrogen bonding is an electrostatic interaction. Therefore, the molecular electrostatic potential (ESP) were calculated using density functional theory based on the ground state geometries of **H‐Pt**, **tBu‐Pt**, and **F‐Pt**. As shown in the ESP maps (**Figure** **3**), complex **H‐Pt** displays electronegative spots (−0.041 to −0.046 a.u.) around the Pt center and the unchelated N atom and electropositive spots (0.039 a.u.) around the hydrogen atoms of pyrimidine. Complex **tBu‐Pt** shows the ESP map similar to **H‐Pt**, and thereby the angles between the centroid distance and the vertical distance in **H‐Pt** dimer (49.8°) and **tBu‐Pt** dimer (49.3°) are comparable. However, compared with that in **H‐Pt**, the unchelated N atom in **tBu‐Pt** shows the stronger electronegativity, which will cause the stronger molecular aggregation. As for **F‐Pt**, the incorporation of F atoms shows notable influence on the ESP by significantly enhancing the electronegativity (−0.060 a.u.) around the unchelated N atom and F atom at 6 position of the phenyl ring and reducing electronegativity (−0.035 a.u.) around the Pt center. The F atom has also totally changed the ESP around 4 position of the phenyl ring from electropositive (0.008 a.u.) in **H‐Pt** to strong electronegative (−0.030 a.u.) in **F‐Pt**, which will not only enhance the strength of the molecular aggregation but also affect the molecular packing orientation by reducing the angle between the centroid distance and the vertical distance in the **F‐Pt** dimer to 28.5°. Besides, the hydrogen atoms of pyrimidine in **F‐Pt** are more electropositive than those in **H‐Pt** and **tBu‐Pt**. Therefore, among **H‐Pt**, **tBu‐Pt,** and **F‐Pt**, the strongest hydrogen bonding interaction with small angle between the centroid distance and the vertical distance in **F‐Pt** dimers can be expected.

**Figure 3:**
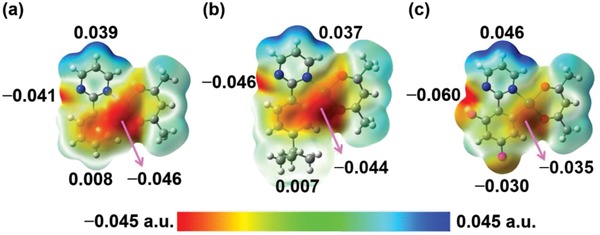
ESP maps of a) **H‐Pt**, b) **tBu‐Pt**, and c) **F‐Pt**.

The room‐temperature UV–vis absorption and phosphorescence spectra of these Pt(II) complexes in CH_2_Cl_2_ are shown in **Figure** **4**, and the related data is listed in **Table** **1**. These complexes exhibited similar absorption spectra in solutions. The high energy transitions with intense absorption bands in the range of 250–320 nm are assigned to the spin‐allowed π–π* transitions, and the weak absorption in the lower‐energy region are ascribed to a mixture of spin allowed singlet metal‐to‐ligand charge transfer (^1^MLCT), spin‐forbidden ^3^π–π* and ^3^MLCT transitions.^44^ In CH_2_Cl_2_, **H‐Pt** and **tBu‐Pt** displayed similar phosphorescence spectra with peaks around 486 and 515 nm, which was comparable with the emission peaks of (2‐phenylpyridinato‐*N*,*C^2′^*)(2,4‐pentanedionato‐*O*,*O*) [(ppy)Pt(acac)] in 2‐methyltetrahydrofuran solution.^44^ The PLQYs of **H‐Pt** and **tBu‐Pt** in degassed THF solutions were estimated to be 0.54 and 0.68, respectively. Because of the incorporation of electron‐withdrawing F atoms, the emission peaks of **F‐Pt** were blue‐shifted by ≈20 nm compared with those of **H‐Pt** and **tBu‐Pt**, which was in good agreement with the blue‐shifted low energy absorption (Figure 4a). However, the vacuum‐deposited neat films of these complexes exhibited distinct different photophysical properties compared with corresponding solutions. The neat film of **H‐Pt** showed two main emission bands. One emission band was located in the blue‐green region, which was very similar to the emission peak of **H‐Pt** in CH_2_Cl_2_ solution, indicating that the emission originated from the monomer; the other emission band was broad and featureless with the peak located at ≈620 nm. The neat films of **tBu‐Pt** and **F‐Pt** also exhibited broad and featureless PL bands with a full width at half maximum over 150 nm and emission maxima at 708 and 727 nm, respectively. The neat film PLQYs of **H‐Pt**, **tBu‐Pt**, and **F‐Pt** were measured as 0.24, 0.55, and 0.74, respectively. The lifetimes were 247, 107, and 314 ns for **H‐Pt**, **tBu‐Pt** and **F‐Pt** in neat films, respectively. As a comparison, the neat film of the pyridine‐based complex (ppy)Pt(acac) showed an emission peak at 605 nm (see Figure S1, Supporting Information) with the PLQY of 0.52, and τ_p_ of 520 ns. The red‐shifted emissions of neat films are indicative of the excimeric and/or MMLCT character.^9^, ^10^, ^41^, ^45^, ^46^, ^47^, ^48^ As revealed by the X‐ray diffraction analysis, the small plane‐to‐plane separation of ≈3.31 Å for **H‐Pt** and ≈3.22 Å for **tBu‐Pt** indicate the strong π–π interaction, and the large *d*_Pt–Pt_ of 4.663(4) Å for **H‐Pt** and 4.8920(8) Å for **tBu‐Pt** confirm the absence of Pt‐Pt interaction, therefore, the bright emissions peaking at 620 and 708 nm for **H‐Pt** neat film and **tBu‐Pt** neat film, respectively, can be ascribed to the excimeric emissions. Because the formation of excimers could quench the luminescence,^49^, ^50^, ^51^ thus **H‐Pt** and **tBu‐Pt** in neat films showed decreased PLQYs compared with those in solutions. However, the *d*_Pt–Pt_ of 3.3752(7) Å is small enough to result in significant Pt–Pt interaction, thus the greatly red‐shifted emission of **F‐Pt** neat film could be induced by the MMLCT transition.^48^ As a result, the PLQY of **F‐Pt** in neat film was significantly increased compared with that of **F‐Pt** in solution due to the MMLCT transition.^9^ Nevertheless, both excimeric and MMLCT characters are related to the enhanced molecular aggregations induced by the intermolecular hydrogen bonds. Considering these pyrimidine‐based complexes have molecular configuration and size similar to (ppy)Pt(acac), the enhanced molecular aggregations and Pt–Pt interactions should result from intermolecular hydrogen bonds. Consequently, with more and stronger hydrogen bonds induced by F atoms, the **F‐Pt** neat film possessed the strongest molecular aggregations and Pt–Pt interactions (supported by the smallest *d*_π–π_ and *d*_Pt–Pt_ in crystal), and thereby showed the longest emission wavelength in the NIR region. This result provides a very simple and promising way to develop efficient NIR emitters. Since NIR light can be generated by excimeric and/or MMLCT transitions in square‐planar Pt(II) complexes, and excimeric and/or MMLCT transitions can be strengthened by enhancing molecular aggregations and Pt–Pt interactions, therefore, we can introduce intermolecular hydrogen bonds into the system with F atoms to enhance the molecular aggregations and Pt–Pt interactions, and then lower the transition energy to effectively generate NIR emissions.

**Figure 4:**
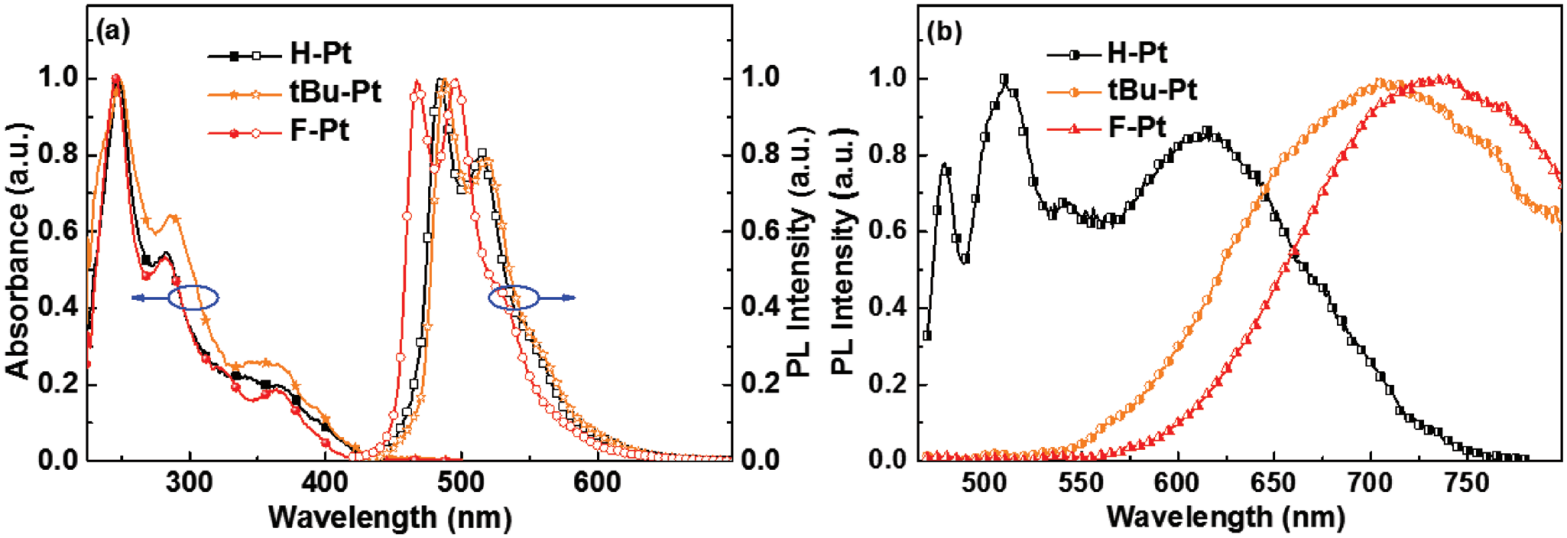
a) Absorption and emission spectra of these Pt(II)complexes in CH_2_Cl_2_, and b) emission spectra of these Pt(II)complexes in neat films.

**Table 1:** Photophysical and electrochemical data for **H‐Pt**, **tBu‐Pt**, and **F‐Pt**

	λ_abs_ a) [nm] (logε)	λ_em_ [nm] CH_2_Cl_2_ a) /filmb)	PLQY THFa) /filmb)	τ_p_ [ns] CH_2_Cl_2_ a) /filmb)	HOMO/LUMOc) [eV]
**H‐Pt**	248 (4.81), 284 (4.55), 369 (4.11)	484, 513/513, 620	0.54/0.24	232/247	−5.18/−2.49
**tBu‐Pt**	248 (4.75), 286 (4.56), 369 (4.14)	488, 518/708	0.68/0.55	218/107	−5.17/−2.50
**F‐Pt**	246 (4.88), 282 (4.60), 363 (4.14)	467, 496/727	0.11/0.74	266/314	−5.28/−2.56

^a)^ The λ_abs_ and λ_em_ were measured in CH_2_Cl_2_ at a concentration of 2 × 10^−5^ m; the PLQY was measured in degassed THF relative to *fac*‐[Ir(ppy)_3_] (PLQY = 0.97); the τ_p_ was recorded in CH_2_Cl_2_

^b)^ Measured in neat films

^c)^ Estimated from the oxidation/reduction peak potentials.

Encouraged by their efficient NIR emissions, neat films of these Pt(II) complexes were used as emitting layers (EMLs) to fabricate OLEDs. Based on our previous study,^9^ the structure of the nondoped device was ITO/TAPC (40 nm)/mCP (8 nm)/neat films of **H‐Pt** (device **A**), **tBu‐Pt** (device **B**) or **F‐Pt** (device **C**) (40 nm)/TmPyPb (30 nm)/LiF (1 nm)/Al (100 nm) in which 1,1′‐*bis*(di‐4‐*tolyl*‐aminophenyl)‐cyclohexane (TAPC), 1,3‐*bis*(carbazol‐9‐yl)benzene (mCP), 1,3,5‐*tri*[(3‐pyridyl)‐phen‐3‐yl]benzene (TmPyPb) and LiF were used as the hole‐injecting layer, hole‐transporting layer, electron‐transporting layer, and electron‐injecting layer, respectively. Driven under suitable bias voltage, these devices showed intense EL with the maxima at 648, 708 and 724 nm for device **A**, **B**, and **C**, respectively (**Figure** **5**). The broad and featureless EL spectra in the low energy region were almost identical to the respective PL spectra of neat films, indicating that the electroluminescence (EL) originated from excimeric and/or MMLCT transitions. It was interesting that weak and discernible emissions in the range from 450 to 500 nm were also observed, which should result from the monomer emissions. However, compared with that of the whole EL spectrum, the integral area of these tiny peaks was negligible, which indicated that these tiny peaks contributed little to the final EQE. The curves of efficiencies versus current density and current density–voltage–radiance (*J*–*V*–*R*) characteristics are shown in Figure 5. Some key data of the OLED devices are summarized in **Table** **2**.

**Figure 5:**
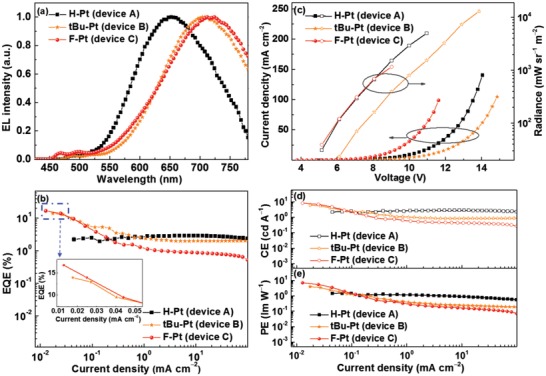
EL characteristics of NIR OLEDs: a) EL spectra, b) curves of EQE versus current density, c) *J–V–R* characteristics, curves of d) CE and e) PE versus current density.

**Table 2:** Key EL performance data of the NIR OLED devices

Device	Emitter	EL λ_max_ [nm]	*V*_turn‐on_ a) [V]	CE_max_ [cd A^−1^]	PE_max_ [cd m^−2^]	EQE_max_ [%]	*R*_max_ [mW sr^−1^ m^−2^]
**A**	**H‐Pt**	648	4.9	2.90	1.46	2.90	5035
**B**	**tBu‐Pt**	708	4.4	6.00	3.87	13.9	10 380
**C**	**F‐Pt**	724	3.8	8.36	6.94	16.7	1164

^a)^ Driving voltage at 1 cd m^−2^.

Device **A** showed a turn‐on voltage of ≈4.9 V, a maximum EQE, current efficiency (CE), power efficiency (PE), and radiance of 2.90%, 2.90 cd A^−1^, 1.46 lm W^−1^, and 5035 mW sr^−1^ m^−2^, respectively. Compared with device **A**, device **B** displayed greatly red‐shifted EL emission at 708 nm with a much higher EQE up to 13.9% and a radiance as high as 10 380 mW sr^−1^ m^−2^. Besides, the peak CE and PE of device **B** were also increasedto 6.00 cd A^−1^ and 3.87 lm W^−1^, respectively. Device **C** achieved the highest EQE of 16.7% with the longest emission wavelength peaking at 724 nm. In addition, the highest peak CE and PE of 8.36 cd A^−1^ and 6.94 lm W^−1^, respectively, were also achieved by device **C**. Although the performance of devices **B** and **C** are inferior to those of the NIR OLEDs reported by Chi very recently, but still outperforms the rest of NIR OLEDs ever reported.^39^, ^40^, ^41^ Considering the very simple synthetic route and outstanding EL efficiencies, pyrimidine‐based Pt(II) complexes **tBu‐Pt** and **F‐Pt** should be ranked among the best NIR emitters so far (see Table S2, Supporting Information). Actually, the device structure was very similar to our previous study,^9^ and were not optimized for these Pt(II) complexes because of our experimental conditions. For example, as listed in Table 1, the highest occupied molecular orbital (HOMO) level of **F‐Pt** was estimated to be −5.28 eV by the cyclic voltammetry (Figure S2, Supporting Information), which did not match well with the HOMO level of mCP (−6.1 eV), resulting in a large hole injection barrier. As for devices based on **tBu‐Pt** and **H‐Pt**, the mismatch of energy levels between the charge transport layers and EML was more serious (HOMO levels for **H‐Pt**, **tBu‐Pt**, and **mCP** are −5.18, −5.17, and −6.1 eV, respectively; lowest unoccupied molecular orbital (LUMO) levels for **H‐Pt**, **tBu‐Pt**, and **TmPyPb** are −2.49, −2.50, and −2.73 eV, respectively), leading to higher driving voltages of devices **A** and **B**. The energy level mismatch might also contribute to efficiencies roll‐off because of the charge imbalance, including the injection process and the transport process.^52^ The device based on **F‐Pt** neat film had smaller injection barriers between the emission layer and the charge transport layers (HOMO levels for **H‐Pt**, **F‐Pt**, and **mCP** are −5.18, −5.28, and −6.1 eV, respectively; LUMO levels for **H‐Pt**, **F‐Pt**, and **TmPyPb** are −2.49, −2.56, and −2.73 eV, respectively), and **F‐Pt** neat film had a higher electron transport ability (see below), therefore, the charge injection/transport behavior within the device based on **F‐Pt** neat film should be more balance than that within the device based on **H‐Pt** neat film at low current density. The balanced charge injection/transport property was one of the main reason that the device based on **F‐Pt** neat film could show the highest EQE, CE, and PE at low current density. However, with the smaller electron injection barrier (Δ*E* = 0.17 eV) and much higher electron transport ability together, the balanced charge transport behavior could not be remained at high current densities, thus leading to efficiencies roll‐off with increased current density. Besides, the long lifetime of neat films may cause serious TTA and thereby the efficiencies roll‐off at high current density.^53^, ^54^ Nevertheless, the PLQYs of these Pt(II) complex neat films are very high, therefore, their EL performance can be further improved if the device structure is painstakingly optimized.

Anyway, devices **B** and **C** are among the best NIR OLEDs in terms of peak EQEs ever reported, and we are more interested in understanding the reasons. Without question, one of the reasons that devices **B** and **C** exhibit much higher EQEs is the high PLQYs of **tBu‐Pt** and **F‐Pt** neat films. In order to further understand the impressive EL performance of devices **B** and **C**, hole‐only and electron‐only devices were fabricated with the structures of ITO/MoO_3_ (3 nm)/*N,N*′‐bis(naphthalen‐1‐yl)‐*N,N*′‐bis(phenyl)benzidine (NPB) (1 nm)/ neat film of Pt(II) complexes (40 nm)/NPB (1 nm)/MoO_3_ (3 nm)/Al (100 nm) and ITO/LiF (3 nm)/1,3,5‐tris(*N*‐phenylbenzimidazole‐2‐yl)benzene (TPBI) (1 nm)/ neat film of Pt(II) complexes (40 nm)/TPBI (1 nm)/LiF (3 nm)/Al (100 nm), respectively. As shown in **Figure** **6**a, hole‐only devices show almost identical current density–voltage (*J*–*V*) curves, implying that these Pt(II) complexes had similar hole transport ability under the same condition. However, under the same driving voltage (> 4 V), the current densities of electron‐only devices based on **tBu‐Pt** and **F‐Pt** were much higher than that of the electron‐only device based on **H‐Pt**, indicating the higher electron transport ability of **tBu‐Pt** and **F‐Pt** neat films than that of **H‐Pt** neat film. The incorporation of fluorine atoms may also benefit the electron injection/transport processes. The strong electron‐withdrawing nature of F atoms would lower the LUMO of **F‐Pt** (LUMO: −2.56 eV for **F‐Pt** vs −2.49 eV for **H‐Pt**) to allow efficient electron injection into **F‐Pt**, and increase the orbital overlap to improve the electronic transfer integral and thereby improving the electron mobility.^55^, ^56^, ^57^ Therefore, **F‐Pt** could show the highest electron mobility. Therefore, device **C** could show more balanced hole and electron transport behavior, and thereby achieve highest EQE.^58^

**Figure 6:**
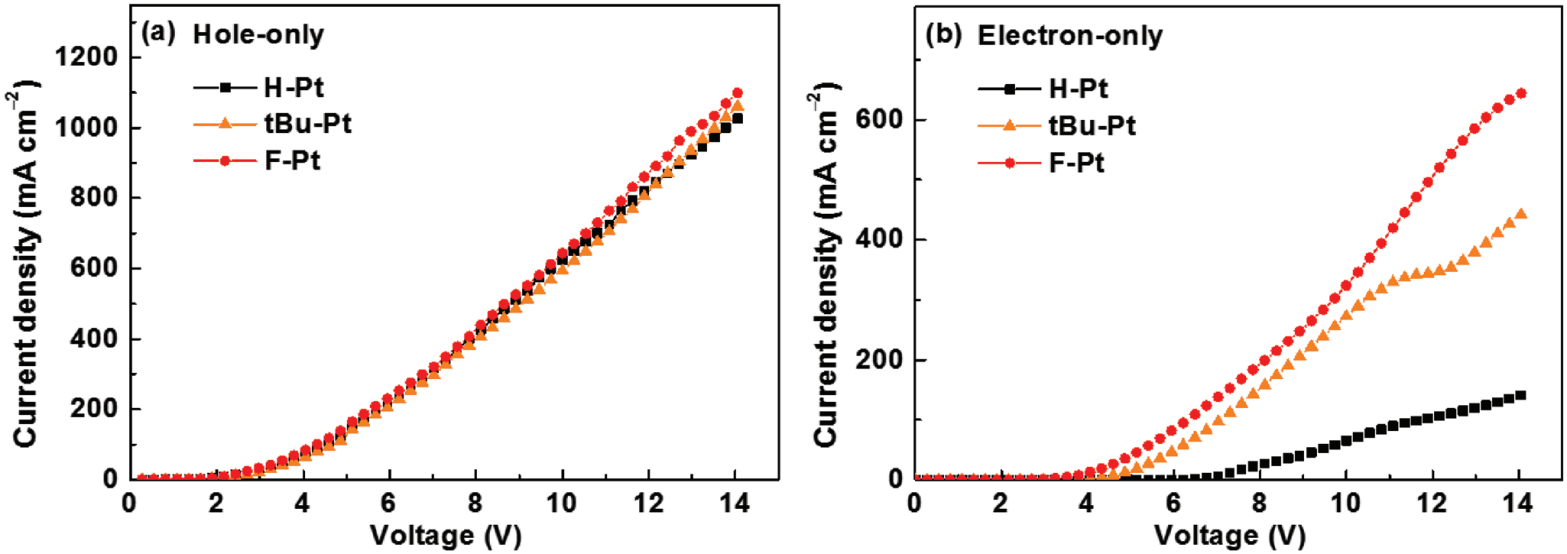
The *J*–*V* curves for a) hole‐only and b) electron‐only devices based on these Pt(II) complex neat films.

Since the molecular orientation and packing pattern have great influences on both charge transport property and device performance, grazing‐incidence wide‐angle X‐ray scattering (GIWAXS) was performed to investigate the molecular orientation and packing behavior in neat films of these Pt(II) complexes. **Figure** **7** shows the 2D GIWAXS patterns of the films. Multiple rings of uniform intensity in Figure 7a indicate that **H‐Pt** molecules have no preferred orientation in the neat film.^59^, ^60^ However, the stronger (100) scattering peaks in the out‐of‐plane direction imply that **tBu‐Pt** and **F‐Pt** have higher degree of crystallinity with more ordered packing behavior in neat films compared with **H‐Pt**. Besides, because of the intermolecular π–π stacking, (010) peaks can be detected at *q*_xy_ = 1.81, 1.83, and 1.85 Å^−1^, corresponding to *d*‐spacings of 3.47, 3.43, and 3.39 Å, for **H‐Pt**, **tBu‐Pt**, and **F‐Pt**, respectively. Obviously, the *d*‐spacing from 2D GIWAXS analysis falls in the order **H‐Pt** > **tBu‐Pt** > **F‐Pt**, which is in good agreement with the order of *d*_π–π_ in single crystals (Figure 1). Therefore, among these Pt(II) complexes in neat films, **F‐Pt** molecules show higher degree of crystallinity with the preferred orientation and stronger π–π intermolecular interaction, which could be ascribed to its more and stronger intermolecular hydrogen bonds induced by F atoms. Consequently, the electron mobility of the **F‐Pt** neat film was much higher than that of the **H‐Pt** neat film, which is consistent with the previous study.^61^ Due to our experimental conditions, we cannot measure the orientation of transition dipole moment by angle‐dependent photoluminescence emission spectroscopy.^62^, ^63^ However, we believe that the GIWAXS results have given us some reliable information about the horizontal orientation of the emission dipoles. As suggested by the sharp and uniform diffraction rings, **H‐Pt** showed a random orientation, indicating its isotropic transition dipole moment vector in neat film. However, **tBu‐Pt** and **F‐Pt**, especially **F‐Pt**, showed more ordered packing behavior in neat films because of the strong intermolecular hydrogen bonds induced by unchelated N atoms and F atoms. Actually, as revealed by the single crystal X‐ray diffraction results, the degree of crystallite orientation also increased from **H‐Pt** to **F‐Pt** (see Figure S3, Supporting Information). The strong intermolecular hydrogen bonds could facilitate **F‐Pt** molecules to form a planar net (Figure 2c). Therefore, **F‐Pt** molecules may have a preferred orientation of transition dipole moments along the horizontal direction, resulting in increased light out‐coupling efficiency and thereby boosting the EQE of device **C**.^8^, ^62^, ^63^, ^64^ In brief, compared with **H‐Pt**, with advantages of higher PLQY, enhanced electron transport ability, and preferred horizontal orientation of transition dipole moments, which all benefit from the strong intermolecular hydrogen bonds induced by F atoms **F‐Pt** neat film could achieve the highest EL efficiency as an EML for the nondoped NIR OLED.

**Figure 7:**
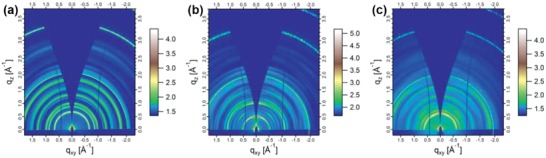
2D GIWAXS images of the vacuum‐deposited neat films of a) **H‐Pt**, b) **tBu‐Pt**, and c) **F‐Pt**.

In summary, three simple pyrimidine‐based Pt(II) complexes had been easily synthesized. As indicated by the single crystal X‐ray diffraction and GIWAXS analysis, compared with **H‐Pt** neat film, **tBu‐Pt** and **F‐Pt** neat films showed higher degree of crystallinity with the preferred molecular orientation because of strong intermolecular hydrogen bonds. Consequently, **tBu‐Pt** and **F‐Pt** neat films displayed significantly red‐shifted emissions in NIR region with peaks at 708 and 727 nm, respectively. Furthermore, **tBu‐Pt** and **F‐Pt** neat films showed high PLQYs of 0.55 and 0.74, respectively. Because of the higher ordered molecular aggregation, **tBu‐Pt** and **F‐Pt** neat films could show significantly enhanced electron transport abilities. With all these advantages, **tBu‐Pt** and **F‐Pt** neat films showed impressive electroluminescent performance with NIR emissions peaking at 708 and 724 nm, respectively. The non‐optimized nondoped NIR OLEDs based on **tBu‐Pt** and **F‐Pt** showed peak EQEs as high as 13.9% and 16.7%, respectively, which were among the highest EQEs for NIR OLEDs ever reported. Through enhancing molecular aggregations with intermolecular hydrogen bonds, this work provides a simple and effective way to develop high‐performance phosphorescent NIR emitters.

[CCDC 1 504 803, 1 504 804, and 1 504 805 contain the supplementary crystallographic data for this paper. These data can be obtained free of charge from The Cambridge Crystallographic Data Centre via www.ccdc.cam.ac.uk/data_request/cif.]

## Conflict of Interest

The authors declare no conflict of interest.

## Supporting information

Supplementary
