# Supplementary material for: Enhancing Molecular Aggregations by Intermolecular Hydrogen Bonds to Develop Phosphorescent Emitters for High‐Performance Near‐Infrared OLEDs
Source: Adv Sci (Weinh). 2019 Feb 7;6(7):1801930. doi: 10.1002/advs.201801930 (PMC6446593; doi:10.1002/advs.201801930)
Supplement: Supplementary file 1 — Supplementary [file ADVS-6-1801930-s001.pdf]

Copyright WILEY-VCH Verlag GmbH & Co. KGaA, 69469 Weinheim, Germany, 2019.

ADVANCED SCIENCE
Open Access

Supporting Information

for *Adv. Sci.,* DOI: 10.1002/advs.201801930

Enhancing Molecular Aggregations by Intermolecular Hydrogen Bonds to Develop Phosphorescent Emitters for High-Performance Near-Infrared OLEDs

Xiaolong Yang, Haoran Guo, Xianbin Xu, Yuanhui Sun, Guijiang Zhou, Wei Ma,* and Zhaoxin Wu**

Copyright WILEY-VCH Verlag GmbH & Co. KGaA, 69469 Weinheim, Germany, 2019.

Supporting Information

Enhancing Molecular Aggregations by Intermolecular Hydrogen Bonds to Develop Phosphorescent Emitters for High Performance Near-Infrared OLEDs

Xiaolong Yang, Haoran Guo, Xianbin Xu, Yuanhui Sun, Guijiang Zhou,* Wei Ma,* and Zhaoxin Wu*

Dr. X. L. Yang, H. R. Guo, Dr. X. B. Xu, Dr. Y. H. Sun, Prof. G. J. Zhou
MOE Key Laboratory for Nonequilibrium Synthesis and Modulation of Condensed Matter
Department of Chemistry
School of Science
Xi'an Jiaotong University
Xi'an 710049, P. R. China
E-mail: zhougj@mail.xjtu.edu.cn

Prof. W. Ma
State Key Laboratory for Mechanical Behavior of Materials
Xi'an Jiaotong University
Xi'an 710049, China
E-mail: msewma@xjtu.edu.cn

Prof. Z. X. Wu
Key Laboratory for Physical Electronics and Devices of the Ministry of Education
Faculty of Electronic and Information Engineering
Xi'an Jiaotong University
Xi'an 710049, P. R. China
E-mail: zhaoxinwu@mail.xjtu.edu.cn

Table of Contents

General information	S3
Synthesis of the complexes	S5
**Table S1.** Crystal data and structure refinement details.	S8
**Figure S1.** Emission spectra of (ppy)Pt(acac) neat film.	S9
**Table S2.** Efficiency of the most advanced NIR OLEDs.	S10
**Figure S2.** CV curves of these pyrimidine-based Pt(II) complexes.	S11
**Figure S3.** Molecular packing pattern in crystals of **H-Pt**, **tBu-Pt** and **F-Pt**.	S11
**References**	S11

General information The raw materials were commercially available and used directly without further purification. All reactions were performed at a nitrogen atmosphere. ^{1}H, ^{19}F and ^{13}C NMR spectra were recorded on a Bruker Avance 400 MHz spectrometer in $CDCl_3$. Chemical shifts were recorded in ppm scale and referenced to the solvent residual peak. Mass spectra (MS) measurements were conducted on a micrOTOF-Q II mass spectroscopy. UV-vis absorption spectra of these pyrimidine-based platinum(II) complexes were measured at room temperature on a Shimadzu UV-2250 spectrophotometer in CH_2Cl_2. Photoluminescence spectra and lifetimes of these complexes were recorded on an Edinburgh Instruments Ltd (FLS920) fluorescence spectrophotometer. The photoluminescence quantum yields (PLQYs) in THF solutions were tested at room temperature against fac-[Ir(ppy)$_3$] standard (Φ_P = 0.97).[1] The PLQYs of neat films were measured with an integrating sphere installed on the FLS920 fluorescence spectrophotometer. Three electrodes including a glassy carbon working electrode, a platinum plate counter electrode, and a platinum wire reference electrode were used to carry out the cyclic voltammetry measurements on the Princeton Applied Research (PARSTAT 2273, Advanced Electrochemical System) equipment in CH_3CN solutions

containing n-Bu_4NPF_6 as the supporting electrolyte with a scan rate of 100 mV s^{-1}, and the ferrocene/ferrocenium (Fc/Fc^+) was used as an internal reference. The HOMO and LUMO energy levels were calculated using the oxidation peak (E_{ox}) and reduction (E_{red}) potentials according to the equations $E_{HOMO} = -(E_{ox} + 4.8)$ eV and $E_{LUMO} = -(E_{red} + 4.8)$ eV.

X-ray Crystallography. Single crystals of these Pt(II) complexes were obtained from $CHCl_3$/hexane solutions by slow evaporation of the solvents. The crystals were mounted on glass fibers and the data were collected on a Bruker SMART CCD diffractometer (Mo Kα radiation and λ = 0.71073 Å) in Φ and ω scan modes at ca. 100 K for **F-Pt**, 150 K for **H-Pt**, and 296 K for **tBu-Pt** 293 K. Their structures were solved by direct methods followed by difference Fourier syntheses, and then refined by full-matrix least-squares techniques against F^2 using SHELXL-97 or SHELXL-2014 program on a personal computer.

GIWAXS measurement. GIWAXS measurements were performed at beamline 7.3.3 at the Advanced Light Source. Samples were prepared on Si substrates by vacuum deposition. The 10 keV X-ray beam was incident at a grazing angle of 0.13°–0.17°, which maximized the scattering intensity from the samples. The scattered X-rays were detected using a Dectris Pilatus 2M photon counting detector.

Theoretical Computation. Theoretical calculation using density functional theory (DFT) was performed for analyzing the molecular electrostatic potentials. Non-metal atoms of C, H, N, O, and F were calculated using B3LYP/6-31G (d, p) basis set. The Pt atom was calculated using B3LYP/LanL2DZ basis set. All calculations were performed by using the Gaussian 09 program. [2]

OLED Fabrication and Measurements. The devices were fabricated by conventional vacuum thermal deposition method under a base pressure around 10^{-3} Pa. Before the deposition of the organic layers, the ITO glass substrates were pre-cleaned with acetone and deionized water, and then exposed to ultraviolet-ozone for ca. 1 min. The thickness of each

layer was determined by a quartz thickness monitor. The brightness–voltage curves of the devices were measured with a Keithley 2602 and Source Meter. The EL spectra and radiance–voltage curves of these devices were recorded with a PR650 SpectraScan spectrometer. All measurements were carried out at room temperature under ambient conditions.

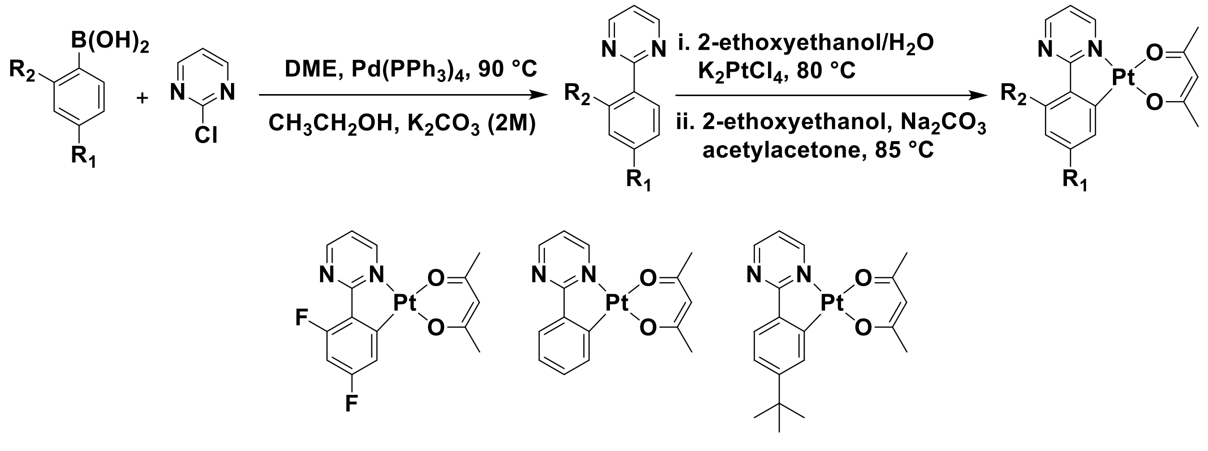

Scheme S1 Synthesis of these pyrimidine-based platinum(II) complexes.

General procedure for synthesizing pyrimidine-based ligands: Under a nitrogen atmosphere, 2-chloropyrimidine (1.0 eqv.), boronic acid compounds (1.0 eqv.) and $Pd(PPh_3)_4$ (0.05 eqv.) were added to a mixture of 1,2-dimethoxyethane (DME, 25 mL), ethanol (5 mL) and K_2CO_3 solution (2M, 20 mL). The reaction mixture was heated to 90 °C and stirred overnight. After cooling to room temperature, the mixture was extracted with CH_2Cl_2 several times. The organic layers were combined, dried over anhydrous $MgSO_4$ and concentrated. The residual was purified on a silica column using a mixture of petroleum ether and ethyl acetate or a mixture of petroleum ether and CH_2Cl_2 as the eluent to give the pyrimidine-based ligands.

2-(2,4-difluorophenyl)pyrimidine (White solid, 86.2% yield). ^{1}H NMR (400 MHz, $CDCl_3$, δ): 8.85 (d, J = 4.8 Hz, 2H), 8.11 (dd, J = 8.4, 6.8 Hz, 1H), 7.24 (t, J = 5.2 Hz, 1H),

7.02–7.6.92 (m, 2H); ^{13}C NMR (100 MHz, $CDCl_3$, δ): 157.27, 133.21, 133.18, 133.11, 133.08, 119.14, 111.72, 111.68, 111.51, 111.47, 105.31, 105.06, 104.80; ^{19}F NMR (376 MHz, $CDCl_3$, δ): –106.76 (d, J = 9.78 Hz, 1F), –110.02 (d, J = 10.15 Hz, 1F).

2-phenylpyrimidine (Color-less solid, 95.4% yield). ^{1}H NMR (400 MHz, $CDCl_3$, δ): 8.81 (d, J = 4.8 Hz, 2H), 8.46–8.43 (m, 2H), 7.52–7.48 (m, 3H), 7.19 (t, J = 5.2 Hz, 1H); ^{13}C NMR (100 MHz, $CDCl_3$, δ): 164.69, 157.22, 137.48, 130.76, 128.59, 128.08, 119.07.

2-(4-(tert-butyl)phenyl)pyrimidine (Color-less solid, 94.3% yield). ^{1}H NMR (400 MHz, $CDCl_3$, δ): 8.79 (d, J = 4.8 Hz, 2H), 8.37 (dd, J = 8.4, 1.6 Hz, 2H), 7.52 (d, J = 8.4 Hz, 2H), 7.15 (t, J = 4.8 Hz, 1H), 1.38 (s, 9H); ^{13}C NMR (100 MHz, $CDCl_3$, δ): 164.74, 157.15, 154.13, 127.90, 126.62, 125.57, 118.75, 34.84, 31.22.

General procedure for synthesizing pyrimidine-based Pt(II) complexes: Under a nitrogen atmosphere, the pyrimidine-based ligand (1.2 eqv.) and K_2PtCl_4 (1.0 eqv.) were added to a mixture of 2-ethoxyethanol and water (3:1, v/v, 35 mL). The reaction mixture was stirred at 80 °C for ca.12 h. After the reaction mixture was cooled to room temperature, colored precipitate was formed by pouring the reaction mixture into water (30 mL). Then the precipitate was filtered and dried. Without further purification, the precipitate was dissolved in 2-ethoxyethenol (30 mL) containing Na_2CO_3 (10.0 eqv.) and acetylacetone (5.0 eqv.). Then the mixture was stirred for ca. 12 h at 85 °C under a nitrogen atmosphere. After the reaction mixture was cooled to room temperature, colored precipitate was formed by pouring the reaction mixture into water (30 mL). Then the precipitate was filtered, dried and purified on preparative TLC plates using petroleum ether and ethyl acetate or a mixture of petroleum ether and CH_2Cl_2 as the eluent to obtain the target pyrimidine-based Pt(II) complexes.

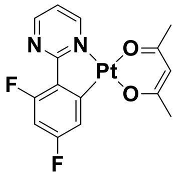

F-Pt

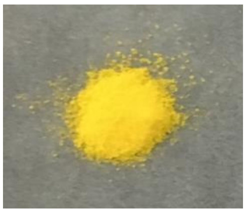

F-Pt (56.8% yield).) MS (EI): m/z 485. ^{1}H NMR (400 MHz, $CDCl_3$, δ): 9.14 (d, J = 5.6 Hz, 1H), 8.90 (s, 1H), 7.15–7.10 (m, 2H), 6.62 (t, J = 10.0 Hz, 1H), 5.51 (s, 1H), 2.03 (s, 3H), 2.01 (s, 3H); ^{13}C NMR (100 MHz, $CDCl_3$, δ): 186.19, 184.64, 158.02, 153.70, 142.98, 116.58, 112.36, 112.20, 102.84, 100.52, 100.27, 100.00, 28.03, 27.00; ^{19}F NMR (376 MHz, $CDCl_3$, δ): –103.13 (d, J = 7.52 Hz, 1F), –110.15 (d, J = 15.04 Hz, 1F).

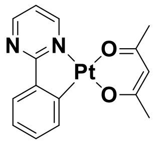

H-Pt

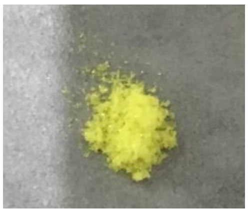

H-Pt (62.5% yield) MS (EI): m/z 449. ^{1}H NMR (400 MHz, $CDCl_3$, δ): 9.10 (dd, J = 5.6, 2.0 Hz, 1H), 8.73 (dd, J = 4.4, 2.0 Hz, 1H), 7.79 (d, J = 6.8 Hz, 1H), 7.63 (d, J = 7.6 Hz, 1H), 7.29 (t, J = 6.8 Hz, 1H), 7.13 (t, J = 7.2 Hz, 1H), 7.06 (t, J = 5.2 Hz, 1H), 5.47 (s, 1H), 2.01 (s, 3H), 1.99 (s, 3H); ^{13}C NMR (100 MHz, $CDCl_3$, δ): 185.87, 184.24, 176.65, 157.79, 153.66, 141.06, 138.34, 131.40, 129.98, 126.72, 123.96, 116.84, 102.58, 28.18, 27.04.

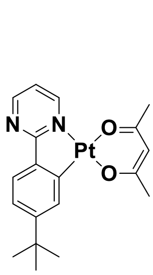

tBu-Pt

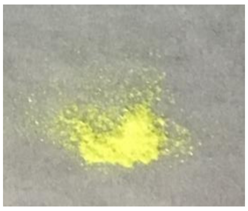

tBu-Pt (60.2% yield) MS (EI): m/z 505. ^{1}H NMR (400 MHz, $CDCl_3$, δ): 9.08 (dd, J = 5.6, 2.0 Hz, 1H), 8.75 (dd, J = 4.4, 2.0 Hz, 1H), 7.71 (d, J = 8.0 Hz, 1H), 7.67 (s, 1H), 7.19 (dd, J = 8.0, 2.0 Hz, 1H), 7.01 (t, J = 5.2 Hz, 1H), 5.48 (s, 1H), 2.03 (s, 3H), 1.99 (s, 3H), 1.38 (s, 9H); ^{13}C NMR (100 MHz, $CDCl_3$, δ): 185.85, 184.16,

176.60, 157.78, 155.08, 153.66, 138.37, 137.82, 126.50, 126.31, 121.58, 116.32, 102.52, 35.25, 31.26, 28.23, 27.11.

Table S1. Crystal data and structure refinement details.

Compound	**F-Pt**	**H-Pt**	**tBu-Pt**
CCDC	1504805	1504804	1504803
formula	$C_{15}H_{12}F_2N_2O_2Pt$	$C_{15}H_{14}N_2O_2Pt$	$C_{19}H_{22}N_2O_2Pt$
M (g mol^{-1})	485.36	505.48	449.37
crystal system	Monoclinic	Monoclinic	Monoclinic
space group	C2/c	P21/n	P21/n
a (Å)	16.716(3)	14.379(2)	13.1018(9)
b (Å)	13.955(3)	8.1884(14)	8.2988(5)
c (Å)	14.240(4)	15.983(3)	13.1051(9)
α (°)	90.00	90.00	90.00
β (°)	120.260(5)	102.112(2)	92.3170(10)
γ (°)	90.00	90.00	90.00
V (Å^3)	2869.1(12)	1840.1(5)	1423.74(16)
Z	8	4	4
D_{calcd} (g cm^{-3})	2.247	1.825	2.096
μ (mm^{-1})	9.811	7.638	9.857
F(000)	1824	976	848
θ range (°)	2.03 to 27.69	2.81 to 27.13	2.16 to 24.81
reflections collected	15745	10123	14243
independent reflections	3329	3958	2450
R_{int}	0.0194	0.0676	0.0202
parameters	201	218	183
R (I > 2σ(I))	R1 = 0.0136, wR2 = 0.0298	R1 = 0.0395, wR2 = 0.1009	R1 = 0.0133, wR2 = 0.0382
R (all data)	R1 = 0.0152, wR2 = 0.0302	R1 = 0.0488, wR2 = 0.1059	R1 = 0.0136, wR2 = 0.0384
S	1.076	1.032	1.265

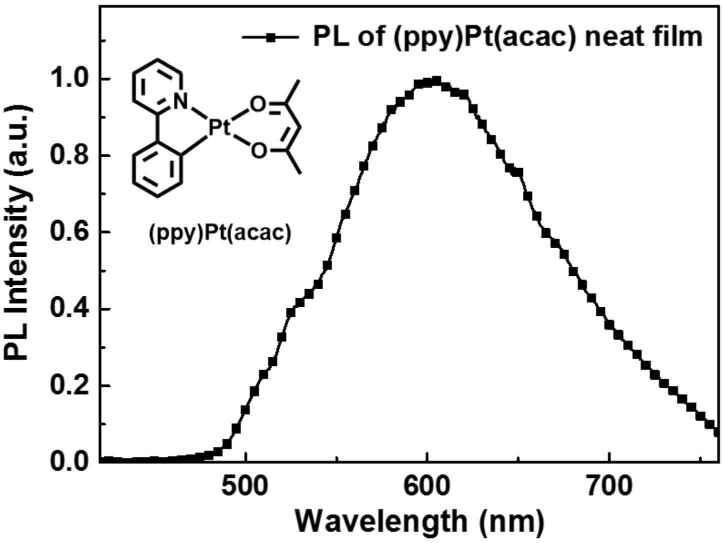

Figure S1. Emission spectrum of (ppy)Pt(acac) neat film.

Table S2. Efficiency of most advanced NIR OLEDs.

Device type	Emitter	λ_{max}^{EL} [nm]	EQE_{max} [%]	Reference
Doped	Polymer	708	0.69	[3]
Doped	Small-Molecule: Polymer Blends	840	1.15	[4]
Non-doped	organic molecule	710	2.1	[5]
Non-doped	organic molecule	741	5.0	[6]
Doped	organic molecule	708	5.53	[7]
Doped	organic molecule	710	7.8	[8]
Doped	organic molecule	700	9.4	[9]
Doped	organic molecule	721	9.74	[10]
Doped	Yb(III) complex	976	0.14	[11]
Doped	Yb(III) complex	976	0.17	[12]
Doped	Os(II) complex	718	2.7	[13]
Doped	Ir(III) complex	780	2.2	[14]
Doped	Ir(III) complex	702	3.4	[15]
Doped	Ir(III) complex	760	4.5	[16]
Non-doped	Pt(II) complex	705	10.5	[17]

Non-doped	Pt(II) complex	700	14.5	[18]
Non-doped	Pt(II) complex	740	24±1	[19]
Doped	Pt(II) complex	769	6.3	[20]
Doped	Pt(II) complex	773	9.2	[21]
Doped	Pt(II) complex	706	6.3	[22]
Doped	Pt(II) complex	704	8.86	[23]

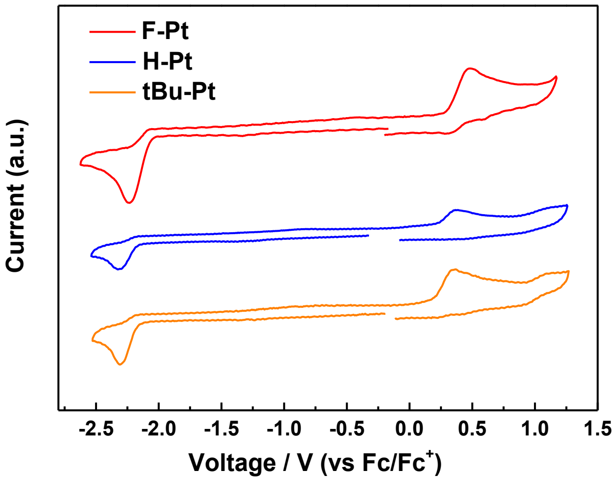

Figure S2. CV curves of these pyrimidine-based Pt(II) complexes.

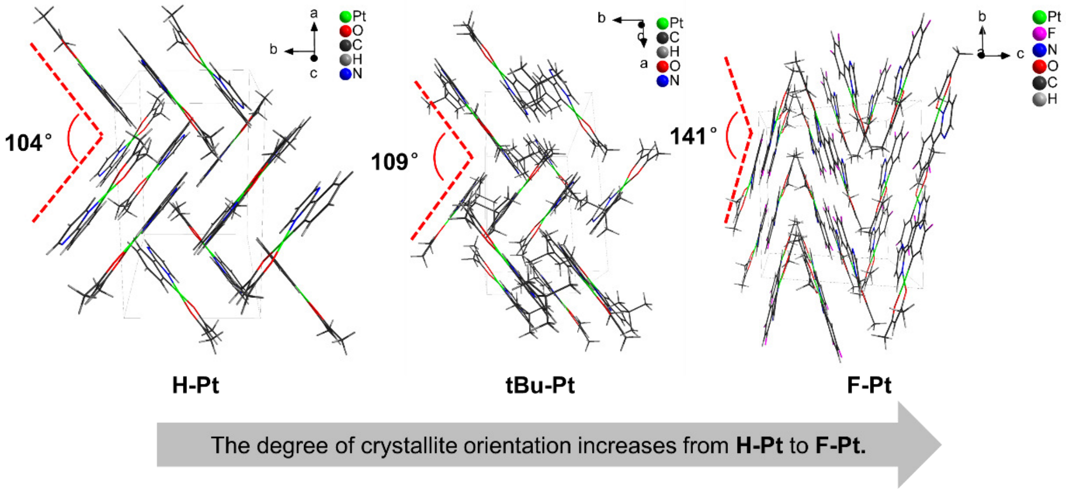

Figure S3. Molecular packing pattern in crystals of **H-Pt**, **tBu-Pt** and **F-Pt**.

References

[1] T. Sajoto, P. I. Djurovich, A. B. Tamayo, J. Oxgaard, W. A. Goddard, M. E. Thompson, J. Am. Chem. Soc. **2009**, 131, 9813.

[2] Gaussian 09, Revision A.02, M. J. Frisch, G. W. Trucks, H. B. Schlegel, G. E. Scuseria, M. A. Robb, J. R. Cheeseman, G. Scalmani, V. Barone, B. Mennucci, G. A. Petersson, H. Nakatsuji, M. Caricato, X. Li, H. P. Hratchian, A. F. Izmaylov, J. Bloino, G. Zheng, J. L. Sonnenberg, M. Hada, M. Ehara, K. Toyota, R. Fukuda, J. Hasegawa, M. Ishida, T. Nakajima, Y. Honda, O. Kitao, H. Nakai, T. Vreven, J. A. Montgomery, J. E. P. Jr., F. Ogliaro, M. Bearpark, J. J. Heyd, E. Brothers, K. N. Kudin, V. N. Staroverov, R. Kobayashi, J. Normand, K. Raghavachari, A. Rendell, J. C. Burant, S. S. Iyengar, J. Tomasi, M. Cossi, N. Rega, J. M. Millam, M. Klene, J. E. Knox, J. B. Cross, V. Bakken, C. Adamo, J. Jaramillo, R. Gomperts, R. E. Stratmann, O. Yazyev, A. J. Austin, R. Cammi, C. Pomelli, J. W. Ochterski, R. L. Martin, K. Morokuma, V. G. Zakrzewski, G. A. Voth, P. Salvador, J. J. Dannenberg, S. Dapprich, A. D. Daniels, Ö. Farkas, J. B. Foresman, J. V. Ortiz, J. Cioslowski, D. J. Fox, Gaussian, Inc., Wallingford CT, **2009**.

[3] X. Gao, B. Hu, G. Tu, Org. Electron. **2014**, 15, 1440.

[4] A. Minotto, P. Murto, Z. Genene, A. Zampetti, G. Carnicella, W. Mammo, M. R. Andersson, E. Wang, F. Cacialli, Adv. Mater. **2018**, 30, 1706584.

[5] S. Wang, X. Yan, Z. Cheng, H. Zhang, Y. Liu, Y. Wang, Angew. Chem. Int. Ed. **2015**, 54, 13068.

[6] P. Data, P. Pander, M. Okazaki, Y. Takeda, S. Minakata, A. P. Monkman, Angew. Chem. Int. Ed. **2016**, 55, 5739.

[7] S. Wang, Y. Miao, X. Yan, K. Ye, Y. Wang, J. Mater. Chem. C **2018**, 6, 6698.

[8] Y. Hu, Y. Yuan, Y.-L. Shi, D. Li, Z.-Q. Jiang, L.-S. Liao, Adv. Funct. Mater. **2018**, 28, 1802597.

[9] C. Li, R. Duan, B. Liang, G. Han, S. Wang, K. Ye, Y. Liu, Y. Yi, Y. Wang, Angew. Chem. Int. Ed. **2017**, 56, 11525.

[10] D.-H. Kim, A. D'Aléo, X.-K. Chen, A. D. S. Sandanayaka, D. Yao, L. Zhao, T. Komino, E. Zaborova, G. Canard, Y. Tsuchiya, E. Choi, J. W. Wu, F. Fages, J.-L. Brédas, J.-C. Ribierre, C. Adachi, Nature Photon. **2018**, 12, 98.

[11] H. Wei, G. Yu, Z. Zhao, Z. Liu, Z. Bian, C. Huang, Dalton Trans. **2013**, 42, 8951.

[12] B. L. Reid, S. Stagni, J. M. Malicka, M. Cocchi, G. S. Hanan, M. I. Ogden, M. Massi, Chem. Commun. **2014**, 50, 11580.

[13] T. C. Lee, J. Y. Hung, Y. Chi, Y. M. Cheng, G. H. Lee, P. T. Chou, C. C. Chen, C. H. Chang, C. C. Wu, Adv. Funct. Mater. **2009**, 19, 2639.

[14] R. Tao, J. Qiao, G. L. Zhang, L. Duan, C. Chen, L. D. Wang, Y. Qiu, J. Mater. Chem. C **2013**, 1, 6446.

[15] X. Cao, J. Miao, M. Zhu, C. Zhong, C. Yang, H. Wu, J. Qin, Y. Cao, Chem. Mater. **2015**, 27, 96.

[16] J. Xue, L. Xin, J. Hou, L. Duan, R. Wang, Y. Wei, J. Qiao, Chem. Mater. **2017**, 29, 4775.

[17] M. Cocchi, D. Virgili, V. Fattori, J. A. G. Williams, J. Kalinowski, Appl. Phys. Lett. **2007**, 90, 023506.

[18] M. Cocchi, J. Kalinowski, D. Virgili, J. A. G. Williams, Appl. Phys. Lett. **2008**, 92, 113302.

[19] K. T. Ly, R.-W. Chen-Cheng, H.-W. Lin, Y.-J. Shiau, S.-H. Liu, P.-T. Chou, C.-S. Tsao, Y.-C. Huang, Y. Chi, Nature Photon. **2017**, 11, 63.

[20] C. Borek, K. Hanson, P. I. Djurovich, M. E. Thompson, K. Aznavour, R. Bau, Y. Sun, S. R. Forrest, J. Brooks, L. Michalski, J. Brown, Angew. Chem. Int. Ed. **2007**, 46, 1109.

[21] K. R. Graham, Y. Yang, J. R. Sommer, A. H. Shelton, K. S. Schanze, J. Xue, J. R. Reynolds, Chem. Mater. **2011**, 23, 5305.

[22] X. Wu, Y. Liu, Y. Wang, L. Wang, H. Tan, M. Zhu, W. Zhu, Y. Cao, Org. Electron. **2012**, 13, 932.

[23] W. Xiong, F. Meng, H. Tan, Y. Wang, P. Wang, Y. Zhang, Q. Tao, S. Su, W. Zhu, J. Mater. Chem. C **2016**, 4, 6007.
